# Detailed analysis of c-di-GMP mediated regulation of *csgD* expression in *Salmonella typhimurium*

**DOI:** 10.1186/s12866-017-0934-5

**Published:** 2017-02-02

**Authors:** Irfan Ahmad, Annika Cimdins, Timo Beske, Ute Römling

**Affiliations:** 1 Department of Microbiology, Tumor and Cell Biology, Karolinska Institutet, Stockholm, Sweden; 2 Present Address: Department of Molecular Biology, Umeå University, Umeå, Sweden; 3 Department of Allied Health Sciences, University of Health Sciences, Lahore, Pakistan; 4 Present Address: Department of Biology, Laboratory for Microbiology, Philipps-University Marburg, Marburg, Germany

**Keywords:** c-di-GMP, CsgD, GGDEF/EAL domain proteins, rdar morphotype, biofilm formation, *Salmonella typhimurium*

## Abstract

**Background:**

The secondary messenger cyclic di-GMP promotes biofilm formation by up regulating the expression of *csgD*, encoding the major regulator of rdar biofilm formation in *Salmonella typhimurium*. The GGDEF/EAL domain proteins regulate the c-di-GMP turnover. There are twenty- two GGDEF/EAL domain proteins in the genome of *S. typhimurium*. In this study, we dissect the role of individual GGDEF/EAL proteins for *csgD* expression and rdar biofilm development.

**Results:**

Among twelve GGDEF domains, two proteins upregulate and among fifteen EAL domains, four proteins down regulate *csgD* expression. We identified two additional GGDEF proteins required to promote optimal *csgD* expression. With the exception of the EAL domain of STM1703, solely, diguanylate cyclase and phosphodiesterase activities are required to regulate *csgD* mediated rdar biofilm formation. Identification of corresponding phosphodiesterases and diguanylate cyclases interacting in the *csgD* regulatory network indicates various levels of regulation by c-di-GMP. The phosphodiesterase STM1703 represses transcription of *csgD* via a distinct promoter upstream region.

**Conclusion:**

The enzymatic activity and the protein scaffold of GGDEF/EAL domain proteins regulate *csgD* expression. Thereby, c-di-GMP adjusts *csgD* expression at multiple levels presumably using a multitude of input signals.

**Electronic supplementary material:**

The online version of this article (doi:10.1186/s12866-017-0934-5) contains supplementary material, which is available to authorized users.

## Background

Bacteria have the ability to live as free-swimming planktonic cells or in the form of multicellular communities known as biofilms, the life style that confers tolerance towards challenging environmental conditions [1, 2]. The ubiquitous second messenger bis- (3′–5′)-cyclic dimeric GMP (c-di-GMP), plays a major role in the transition from the motile to the sessile life style on the single cell level [3–6]. C-di-GMP is synthesized by diguanylate cyclases (DGCs), GGDEF domain proteins [7–10], and degraded by c-di-GMP phosphodiesterases (PDEs), EAL or HD-GYP domain proteins [11–13]. These cyclic di-GMP metabolizing proteins occur almost ubiquitously in bacterial genomes with single bacterial genomes to possess from a single GGDEF/EAL domain protein to more than hundred [6, 14–16]. Elucidating the precise role of individual GGDEF/EAL domain proteins will contribute to the understanding of the complex regulation of bacterial physiology by the c-di-GMP signalling system.

A variety of phenotypes such as motility, cell cycle and differentiation and virulence are regulated by c-di-GMP signalling, however, biofilm formation is studied most extensively [17–19]. In the model organism *Salmonella enterica* serovar Typhimurium UMR1, c-di-GMP promotes a rdar (**r**ed, **d**ry **a**nd **r**ough) biofilm formation by stimulating the production of the extracellular matrix components, the exopolysaccharide cellulose and proteinaceous curli fimbriae [20, 21].

Expression of the rdar morphotype is regulated by the LuxR family transcriptional activator CsgD, a major hub in rdar biofilm formation in *S. typhimurium* [22, 23]. CsgD is central in regulating the transition between biofilm formation and virulence. *csgD* expression is usually regulated by environmental stimuli such as temperature and growth phase from the transcriptional to the posttranscriptional level [24]. Global transcriptional regulators such as RpoS, OmpR, H-NS and IHF regulate the transcription of *csgD* in *S. typhimurium* [25]. *CsgD* expression is also adjusted post-transcriptionally by several small sRNAs and the RNA chaperone Hfq [26–28] and is a major target of c-di-GMP signalling [20, 29].

The genome of *S. typhimurium* contains twenty-two GGDEF/EAL domain proteins, not all are bona fide c-di-GMP metabolizing proteins [20, 30]. Task distribution is shown as distinct panels of proteins are associated with specific phenotypes such as *csgD* expression, cellulose biosynthesis, motility, invasion of epithelial cells, stimulation of a pro-inflammatory immune response and colonization of the gastrointestinal tract of mice [20, 30].

In rdar biofilm formation, two GGDEF-EAL proteins, STM3388 and STM2123 promote, while the four EAL domain proteins STM1703, STM4264, STM3611 and STM1827 suppress *csgD* expression [20, 31]. The transcriptional regulator CsgD activates the expression of *csgBA*, encoding the minor and major subunit of curli and *adrA*, encoding the diguanylate cyclase AdrA. C-di-GMP produced by AdrA stimulates the cellulose synthase in order to activate cellulose biosynthesis [20, 32].

To further dissect the network of GGDEF and EAL domain proteins, we identified two novel GGDEF domain proteins to regulate *csgD* expression. Deletion of major phosphodiesterases indicates that elevated c-di-GMP levels regulate *csgD* expression and rdar morphotype by multiple pathways. Identification of corresponding diguanylate cyclases and phosphodiesterases points to local and global regulation of *csgD* expression by c-di-GMP signalling.

## Methods

### Bacterial strains, plasmids, and growth conditions

Bacterial strains and plasmids are listed in Additional file 1. For cloning purposes, *E. coli* TOP10 and *S. typhimurium* were grown on Luria-Bertani (LB) agar plates supplemented with appropriate antibiotics. Otherwise, bacteria were pre cultured on LB agar plates at 37°C overnight and directly inoculated on LB agar plates without salt. Antibiotics were ampicillin (100 μg ml^−1^), chloramphenicol (20 μg ml^−1^), kanamycin (30 μg ml^−1^), and tetracycline (20 μg ml^−1^). For expression of genes, 0.1% arabinose and 1 mM IPTG was used.

### Construction of *S. typhimurium* mutants

The deletion mutant of *ompR* was created by one-step gene inactivation [33]. Entire open reading frame except 40 nucleotides at the beginning and at the end of the gene were replaced by a chloramphenicol resistance marker. Approximately 300 ng of processed PCR product amplified from pKD3 or pKD4 was electroporated into *S. typhimurium* UMR1 containing pKD46. Recovered colonies were purified at least twice on LB medium containing appropriate antibiotics.

Mutant alleles were combined by phage transduction using phage P22 HT105/1 *int-201* whereby the resistance marker of the parent strain was cut out using pCP20 [34]. Transductants were colony purified twice on LB agar plates containing 10 mM EGTA and appropriate antibiotics. All constructed mutants were verified by PCR with control primers located in genes flanking the targeted open reading frame. All quadruple and pentapole mutants were verified after strain construction.

Site directed mutagenesis to replace the glutamate in the EAL motif of STM4264 by alanine was carried out by scar less site directed mutagenesis [35]. In brief, a chloramphenicol resistance cassette fused to an I-*Sce*I recognition site by homologous recombination replaced the codon for glutamic acid 303 of STM4264. Subsequently, plasmid pWRG99, which encodes I-*Sce*I endonuclease under a tetracycline inducible promoter, aided replacement of the chloramphenicol cassette by a DNA fragment of 80 bp (containing the E303A mutation obtained by annealing primers ‘4264-303A-mut-scarless forw’ and ‘4264-E303A-mut-scarless Rev’) after selection on LB agar plates with IPTG, arabinose and tetracycline [35]. STM4264 harboring the desired mutation was verified by DNA sequencing. Primers used in this study are listed in Additional file 1: Table S2﻿.

### Plasmid construction

Plasmid pBAD30::2123 was constructed by cloning STM2123 into pBAD30 with a C-terminal 6xHis tag. STM2123 was amplified with primers ‘STM2123 cloning Forw’ and ‘STM2123 cloning Rev’ harboring restriction sites *Xba*I and *Hind*III and the restricted DNA fragment was ligated into pBAD30.

### Construction of mutant GGDEF/EAL proteins

To generate mutations in GGDEF and EAL domains mutagenic oligonucleotides were designed (listed in Additional file 1). The QuickChange mutagenesis kit (Agilent Technologies) was used according to the manufacturer’s protocol. The resulting mutations were confirmed by DNA sequencing.

### Rdar morphotype assay

Five microliters of a bacterial suspension in water (OD_600_ of 5) from an overnight culture in LB broth were spotted onto LB without salt agar plates supplemented with Congo red (40 μg ml^−1^) and Coomassie brilliant blue (20 μg ml^−1^). Plates were incubated at 28°C for 48 h. Development of the colony morphology and dye binding was analysed over time.

### Protein techniques

For western blot analysis of CsgD and c-di-GMP turnover protein expression, cells were grown on LB agar plates without salt for 24 h at 28°C. 5 mg (wet weight) cells were harvested, resuspended in 200 μl SDS sample buffer, and incubated at 95°C for 10 min. Membrane proteins were resuspended in sample buffer with 8M Urea. The protein content was analysed by Coomassie blue staining (20% methanol, 10% acetic acid, 0.1% Coomassie brilliant blue G) after sodium dodecyl sulfate-polyacrylamide gel electrophoresis (12% resolving gel and 4% stacking gel). Equal amounts of protein were separated and subsequently transferred to a polyvinylidene difluoride membrane (Immobilon P; Millipore). Detection of CsgD was carried out using a polyclonal anti-CsgD peptide antibody (1:5,000) and detection of 6xHis-tagged proteins with monoclonal anti 6xHis antibody as the primary antibodies and goat anti-rabbit/mouse immunoglobulin G conjugated with horseradish peroxidase (1:2,000; Jackson ImmunoResearch Laboratories Inc.) as the secondary antibodies, respectively [22]. Chemiluminescence (Lumi-Light WB substrate; Roche) was recorded using the LAS-1000 system (FUJIFILM) [25, 36]. Strain *S. Typhimurium* MAE52 was used as a positive control, whereas strain MAE50, a *csgD* deletion mutant, was used as negative control. Western blotted membranes were subjected to the Ponceau S staining to confirm equal loading of protein samples where appropriate.

### Beta galactosidase assay

Promoter activity of *csgD* was assayed with different *csgD* promoter constructs [25, 36]. Expression of *adrA* was analyzed with a chromosomal MudJ transcriptional fusion in *adrA* [37]. Strains were grown on LB without salt plates supplemented with appropriate antibiotics and inducer. Samples were collected after growth for 24 h at 28°C. β-galactosidase activity was the read out for promoter activity [38] after adjustment of bacterial suspension to OD_600_ = 0.4 for pUGE13 and to 0.1 for pUGE5, pUGE7 and pUGE19. Normalized β-galactosidase activity was calculated using the formula: Miller units = 1000 {[OD420 ‐ (1.75 × OD_550_)]/(t × V × OD_600_)} with t = reaction time in min; V = volume of cell suspension. All β-galactosidase measurements were done in duplicates using at least three technical replicates. Statistical analysis was performed applying an unpaired t-test with two-tailed p-value (*** is *p* < 0.0001) using Prism 5 (GraphPad Software).

## Results

### Identification of novel GGDEF domain proteins promoting *csgD* expression

**Fig. 1:**
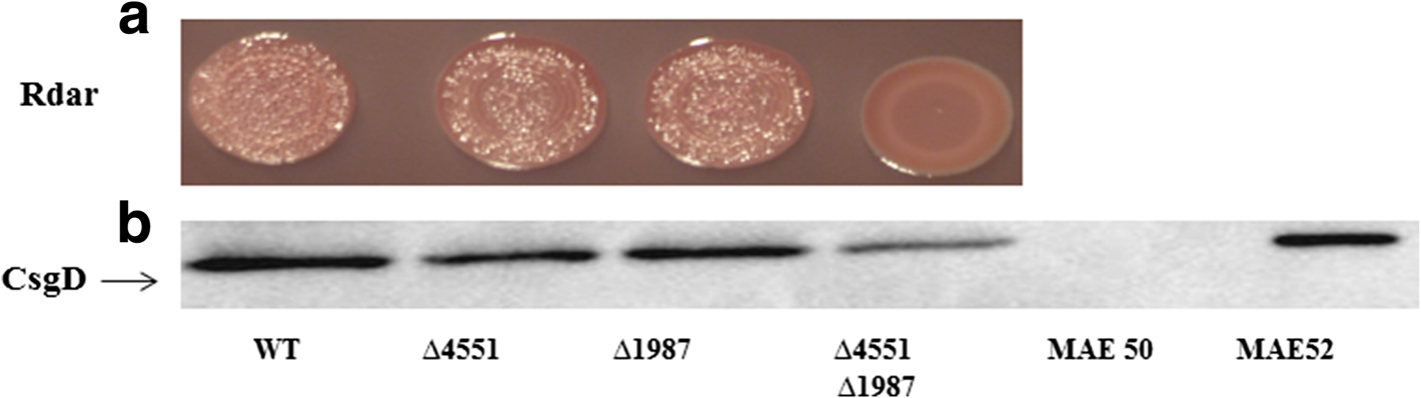
Identification of novel GGDEF domain proteins regulating *csgD* expression. **a** Rdar morphotype formation of *S. typhimurium* UMR1 and STM1987 and STM4551 mutants after 48 h of growth on Congo red agar plates. **b** CsgD levels after 24 h of growth at 28°C on LB without salt agar plates*.* WT is wild type *S. typhimurium* UMR1. MAE50 is a *csgD* deletion mutant of UMR1 (negative control) whereas MAE52 strain is used as a CsgD positive control

Multicellular behavior as expressed by the rdar biofilm morphotype in *S. typhimurium* UMR1 correlates with expression of the response regulator CsgD, a major target of c-di-GMP signalling. As *csgD* expression is not completely abolished in a STM3388 and STM2123 double mutant [20], re-assessment of the effect of the remaining GGDEF proteins identified STM4551 and STM1987 to additionally activate *csgD* mediated rdar morphotype expression. The respective single deletion mutants exhibited a down regulation of the rdar morphotype (Fig. 1a) and CsgD levels (Fig. 1b) and the double mutant had an additive effect (Fig. 1a).

### Additive effect of GGDEF domain proteins on *csgD* expression

**Fig. 2:**
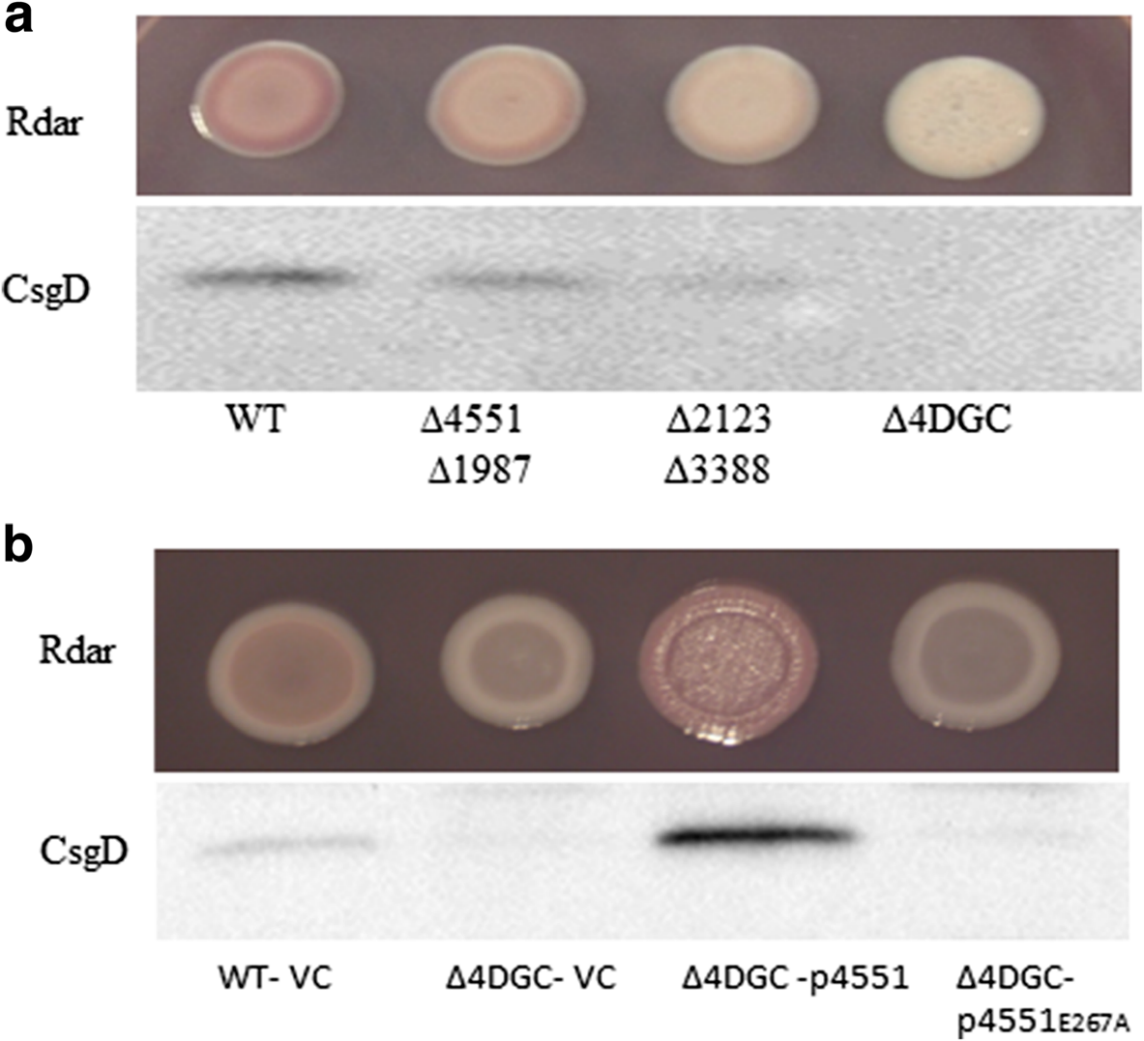
Cumulative effect of GGDEF proteins on rdar morphotype and *csgD* expression in *S. typhimurium* UMR1. **a** Rdar morphotype and CsgD levels of double and quadruple mutants of GGDEF proteins after 24 h of growth at 28°C on LB without salt agar plates. **b** Overexpression of the diguanylate cyclase STM4551 in the Δ4DGC mutant restored rdar morphotype and *csgD* expression, in contrast to catalytically inactive STM4551_E267A_. Cells were grown for 24 h at 28°C on LB without salt agar plates supplemented with ampicillin (100 μg ml^−1^) and 0.1% L-arabinose. VC = Vector control pBAD30, p4551 = STM4551 cloned in pBAD30, p4551_E267A_ = catalytic mutant STM4551_E267A_ cloned in pBAD30

A quadruple mutant with deletion of *STM4551, STM1987, STM3388* and *STM2123* (Δ4DGC) exhibited a smooth and white (saw) morphotype on CR agar plates (Fig. 2a) with CsgD expression additionally down regulated (Fig. 2a). Over expression of the GGDEF protein STM4551 from pBAD30 restored rdar morphotype and *csgD* expression in the Δ4DGC mutant of *S. typhimurium* UMR1. Although STM4551 is an established diguanylate cyclase [30, 39], reportedly its catalytic activity is not required to restore most of the phenotypes associated with the deletion of 12 GGDEF domain proteins in *S. enteritidis* including *csgD* expression [39]. However, a catalytic mutant of STM4551 with the GGDEF motif altered to GGAEF did not affect the smooth and white colony morphotype and *csgD* expression, suggesting that the lack of c-di-GMP in Δ4DGC is the only factor mediating the down regulation of rdar morphotype and *csgD* expression.

### C-di-GMP turnover regulated by GGDEF/EAL domain proteins modulates *csgD* expression

**Fig. 3:**
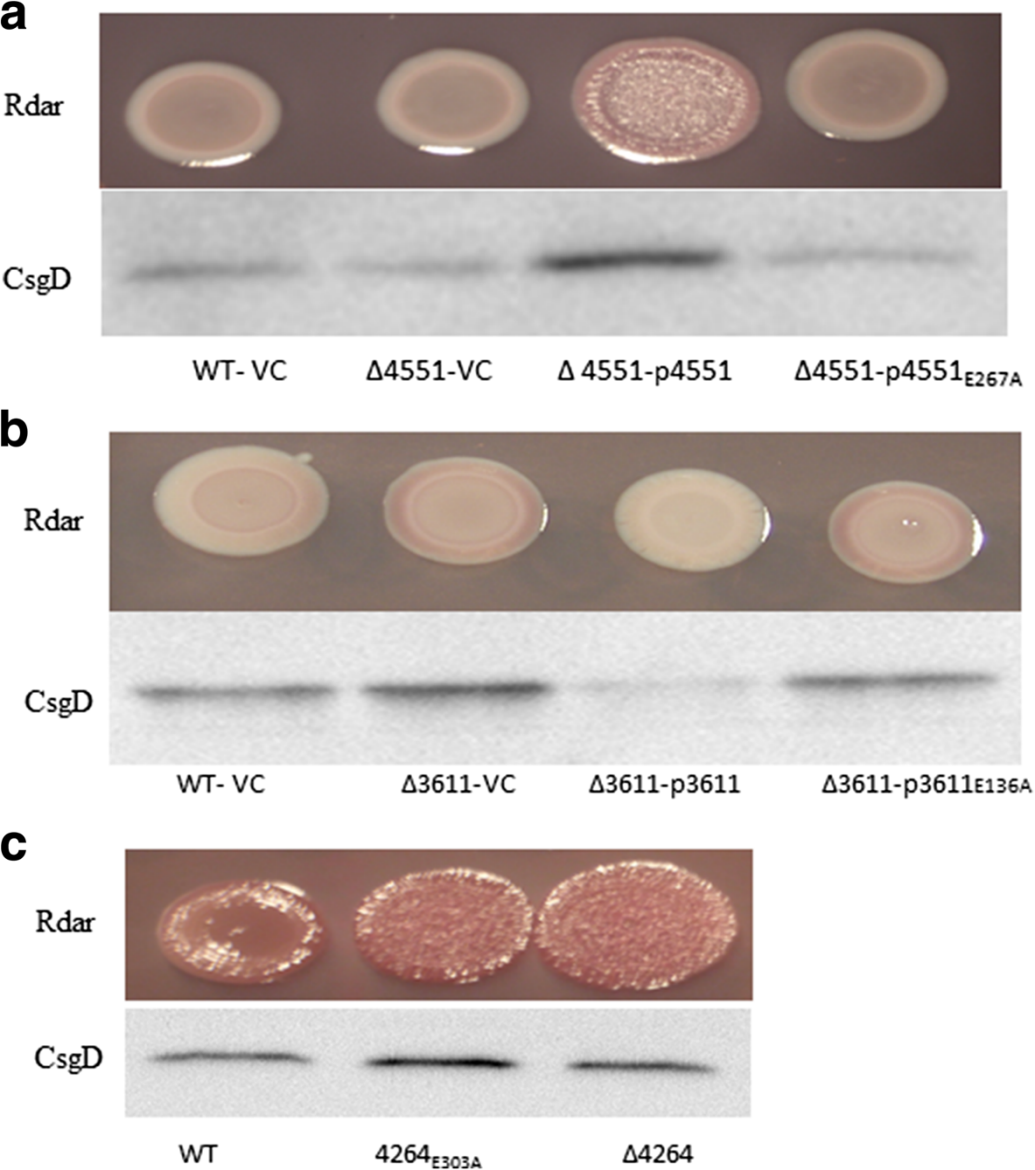
Complementation of the rdar morphotype and *csgD* expression phenotypes of GGDEF/EAL mutants of *S. typhimurium* UMR1. Complementation by the GGDEF protein STM4551 (**a**) and the EAL protein STM3611 (**b**) in respective mutants. Catalytically inactive proteins did not restore *csgD* expression and the rdar morphotype. **c** Rdar morphotype formation and CsgD levels of *S. typhimurium* UMR1 upon the chromosomal replacement of the EAL to AAL motif of EAL protein STM4264. Cells were grown for 24 h at 28°C on LB without salt agar plates supplemented with ampicillin (100 μg ml^−1^) and 0.1% L-arabinose

As c-di-GMP is required for *csgD* expression, the contribution of c-di-GMP metabolism to regulate *csgD* expression was investigated for individual GGDEF and EAL proteins. Two GGDEF proteins STM4551 and STM1987 (Fig. 2a) and two GGDEF-EAL proteins STM2123 and STM3388 ([20] and Fig. 2a) promote *csgD* mediated biofilm formation in *S. typhimurium* UMR1. On the other hand, three EAL domain proteins STM4264, STM3611 and STM1827 and the GGDEF-EAL domain protein STM1703 suppress *csgD* expression [31]. Wild type GGDEF domain proteins along with their catalytically inactive variants were overexpressed in the respective chromosomal mutants to assess the impact of catalytic activity. The GGDEF domain protein STM4551 promoted rdar morphotype and *csgD* expression (Fig. 3a) when overexpressed in the respective mutant strain, whereas the catalytically inactive variant STM4551_E267A_, although expressed at the same level as wild type (data not shown), did not affect rdar morphotype and *csgD* expression.

Overexpression of the EAL proteins STM3611 and STM1827 suppressed rdar morphotype and *csgD* expression in the respective chromosomal mutants (Fig. 3b, Additional file 2: Figure S1), whereas the mutants STM3611_E136A_ and STM1827_E302A_ did not exhibit an altered phenotype although mutant proteins were expressed at the same levels as wild type (data not shown). These findings suggest that the action of STM3611 and STM1827 as suppressors of *csgD* expression is due to their c-di-GMP phosphodiesterase activity. A partial effect of the STM3611 protein scaffold on *csgD* expression cannot be excluded at this stage, though.

**Fig. 4:**
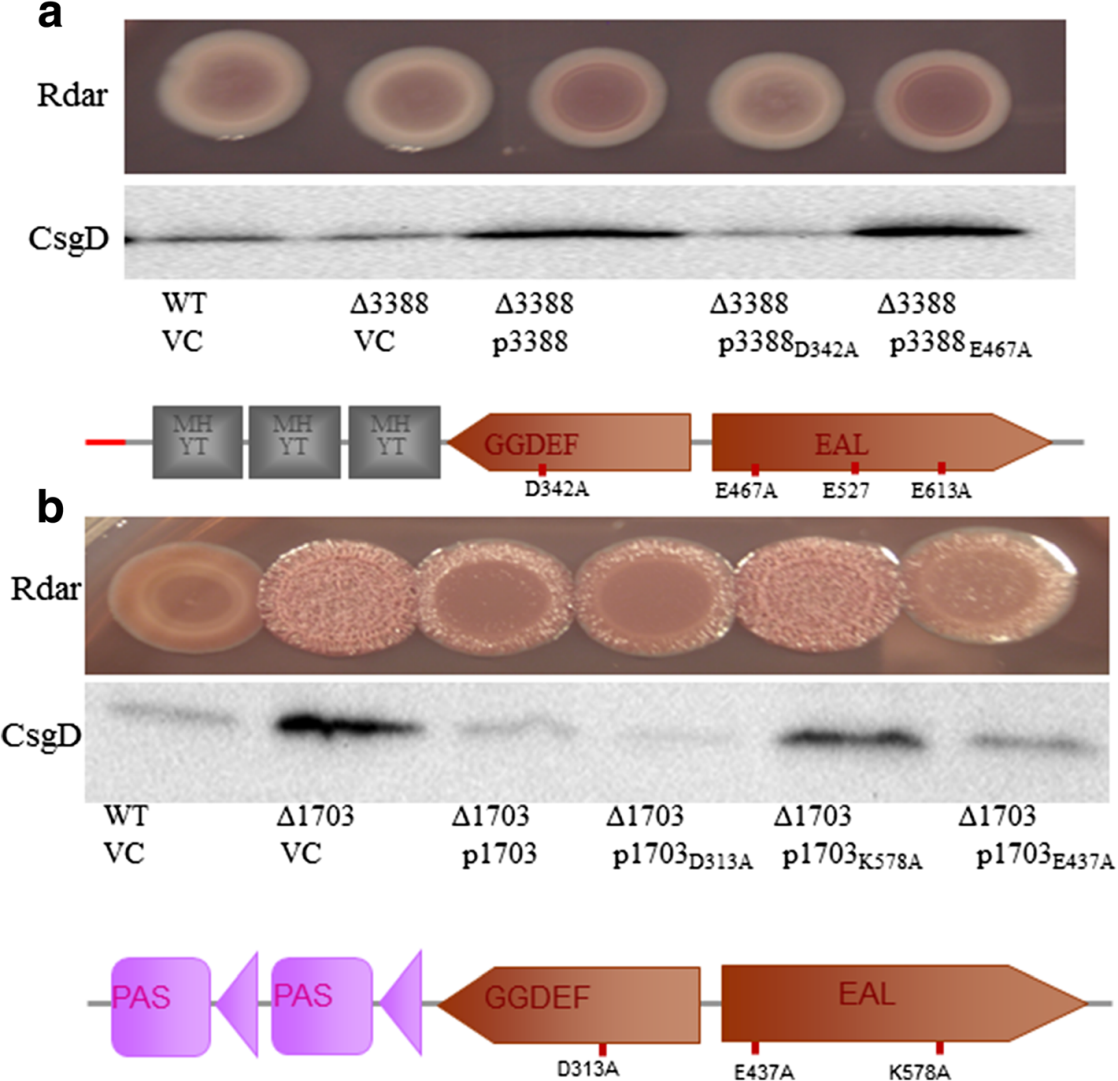
The GGDEF-EAL domain protein STM3388 promotes *csgD* expression through its diguanylate cyclase activity, whereas the GGDEF-EAL domain protein STM1703 suppresses *csgD* expression through its phosphodiesterase activity. **a** Rdar morphotype and CsgD levels upon expression of wild type STM3388 and catalytic GGDEF and EAL mutants of STM3388 in STM3388 deletion mutant of *S. typhimurium* UMR1. **b** Rdar morphotype and CsgD levels upon overexpression of STM1703 and catalytic mutants of STM1703 in the *STM1703* deletion background of *S. typhimurium* UMR1. Cells were grown for 24 h at 28°C on LB without salt agar plates supplemented with ampicillin (100 μg ml^−1^) and 0.1% L-arabinose. VC = Vector control pBAD30

Expression of the GGDEF-EAL domain protein STM3388 from pBAD30 promotes rdar morphotype formation and *csgD* expression (Fig. 4a) due to its diguanylate cyclase activity. The catalytically inactive GGDEF domain mutant STM3388_D342A_ suppressed rdar morphotype and *csgD* expression slightly, suggesting a minor phosphodiesterase activity. Similarly, expression of STM3388_E467A,_ which contains a mutated EAL motif, slightly promotes rdar morphotype and *csgD* expression. Although protein expression data are missing, these results are consistent with reported apparent time dependent diguanylate cyclase and phosphodiesterase activity of the GGDEF-EAL domain protein STM3388 [20]. A deletion mutant of STM3388 showed enhanced *csgD* expression in early growth phase which was diminished later in the growth phase.

The GGDEF-EAL domain protein STM2123 is a phenotypic diguanylate cyclase [20], consistent with bioinformatic analysis, which predicts a catalytically functional GGDEF domain and a non-functional EAL domain [30]. Over expression of STM2123 enhanced *csgD* expression and c-di-GMP levels [20]. However, reassessment of the cloned STM2123 gene revealed a deletion of 10 amino acids at the C-terminus. Expression of full length STM2123 and its GGDEF mutant STM2123_D651A_ and I site mutant STM2123_R640A_ did not promote rdar morphotype and *csgD* expression at different arabinose concentrations up to 0.1% (Additional file 2: Figure S1, data not shown). In summary, at least two GGDEF proteins STM4551 and STM3388 contribute to *csgD* expression through c-di-GMP turnover. The GGDEF protein STM1987 could not be cloned without mutation and was therefore excluded from the analysis.

The GGDEF-EAL protein STM1703 displays apparent phosphodiesterase activity [31], although bioinformatic analysis predicts a catalytically functional GGDEF and EAL domain. A catalytic mutant in the GGDEF motif, STM1703_D313A_, however, displays down regulation of the rdar morphotype compared to the wild type protein suggesting an active diguanylate cyclase (Fig. 4b). Amino acid exchange of EAL to AAL motif abolished phosphodiesterase activity in all EAL domain proteins examined previously [31]. The 1703_E437A_ mutant still down regulates the rdar morphotype and *csgD* expression equally as mutants STM1703_E527A_ and STM1703_E613A_ (data not shown). The catalytic mutants of STM1703 are equally expressed as wild type protein except STM1703_K578A_, which did not show functionality (Fig. 4b and data not shown). Interestingly, upon expression of STM1703_E437A_ in the *S. typhimurium* UMR1 background, rdar morphotype downregulation was not observed, while STM1703_E527A_ and 1703_E613A_ still displayed downregulation of the rdar morphotype (Additional file 2: Figure S2). These findings suggest a complex role of STM1703 in *csgD* regulation dependent on the enzymatic activity and the protein scaffold.

The EAL domain protein STM4264 could not be cloned without mutation. A scar less single amino acid replacement of the glutamate in the EAL motif led to enhanced rdar morphotype and *csgD* expression (Fig. 3c) similar to the deletion mutant of STM4264. Taken together, these findings indicate that the phosphodiesterase activities of STM4264, STM3611 and STM1827 are required to suppress rdar morphotype formation and *csgD* expression while the situation with respect to STM1703 is more complex. The GGDEF and EAL proteins and respective catalytic mutants are summarized in Additional file 2: Figure S6.

### Modulation of *csgD* expression by a complex network of GGDEF/EAL domain proteins

**Fig. 5:**
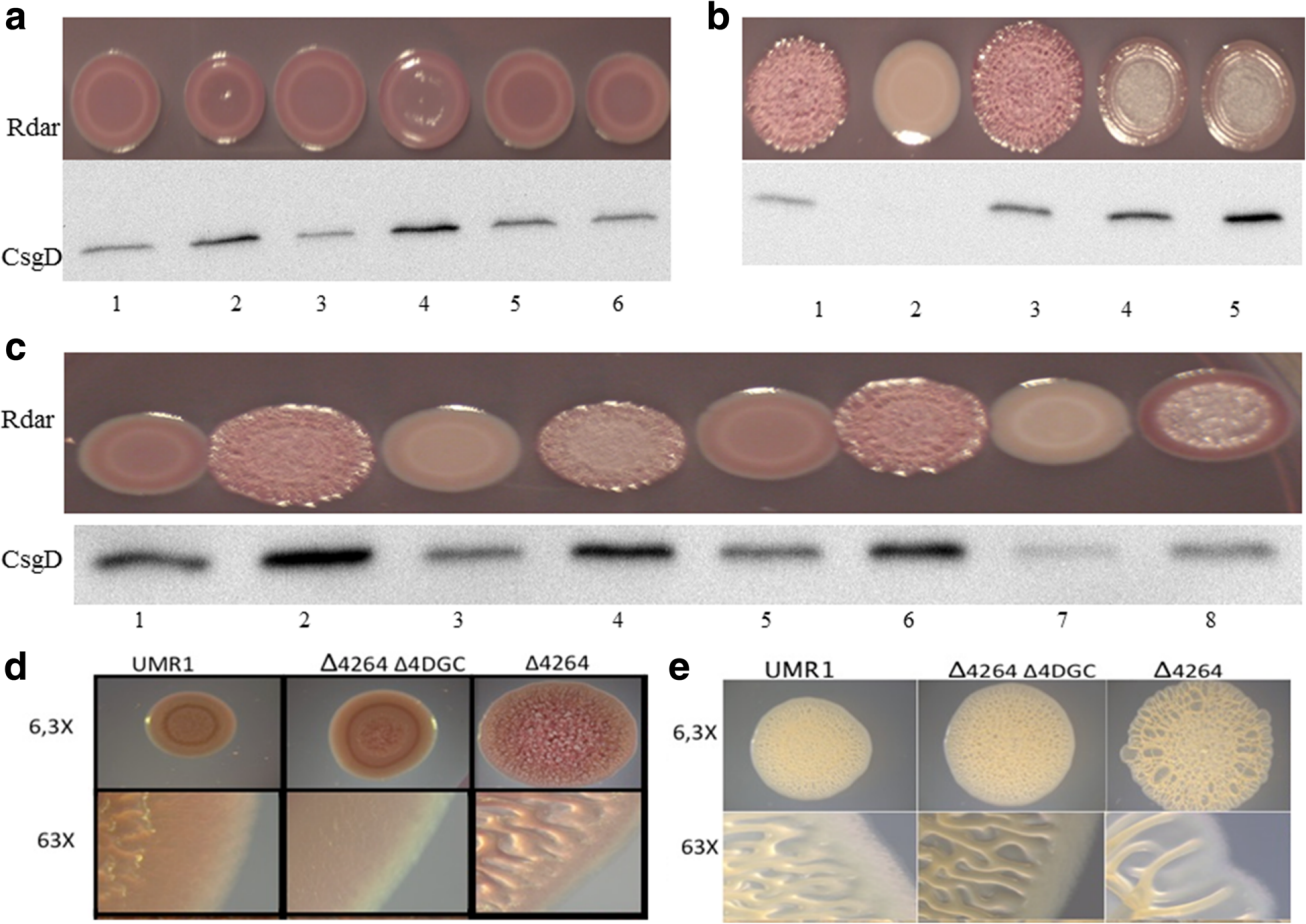
Corresponding GGDEF/EAL domain proteins. Rdar morphotype and CsgD levels of *S. typhimurium* UMR1 upon deletion of the EAL domain protein STM3611 (**a**), the GGDEF-EAL protein STM1703 (**b**), and the EAL protein STM4264 (**c**) in different diguanylate cyclase mutant backgrounds. Alteration in rdar morphotype upon *adrA* deletion is due to lack of cellulose synthesis. Stereomicroscopic image of the rdar morphotype formation of the STM4264 mutant upon the deletion of four diguanylate cyclases after 48 h of growth at 28°C on LB without salts plates supplemented with Congo red (**d**) and without Congo red (**e**). Captions (**a**) 1 = UMR1Δ2123, 2 = UMR1Δ3611Δ2123, 3 = UMR1Δ3388, 4 = UMR1Δ3611Δ3388, 5 = UMR1Δ4551Δ1987, 6 = UMR1Δ3611Δ4551Δ1987. **b** 1 = UMR1Δ1703, 2 = UMR1Δ4551Δ1987Δ2123Δ3388, 3 = UMR1Δ1703Δ4551 Δ1987Δ2123Δ3388, 4 = Δ1703ΔAdrA 5 = Δ1703ΔAdrAΔ1283Δ2672. **c** 1 **=** UMRI, 2 = UMR1Δ4264, 3 = UMR1 Δ2123Δ3388, 4 = UMR1Δ4264Δ2123Δ3388, 5 = UMR1Δ4551Δ1987. 6 = UMR1Δ4264Δ4551Δ1987, 7 = UMR1Δ4551Δ1987Δ2123Δ3388, 8 = UMR1Δ4264Δ4551Δ1987Δ2123Δ3388

Occurrence of multiple diguanylate cyclases and phosphodiesterases dedicated to *csgD* regulation raises the question whether these proteins operate in specific combination i.e.: Do specific phosphodiesterases degrade c-di-GMP synthesized by specific diguanylate cyclases? To identify corresponding diguanylate cyclases and phosphodiesterases, EAL proteins were deleted in the background of GGDEF deletion mutants. If the deleted phosphodiesterase is solely or mainly required for degrading c-di-GMP produced by the deleted diguanylate cyclase, no increase in *csgD* expression is observed. Enhancement of *csgD* expression upon phosphodiesterase deletion in the strain background of deleted diguanylate cyclase indicates no counteraction of the c-di-GMP pool. Deletion of STM3611 in the STM3388 and STM2123 mutants enhanced rdar morphotype and *csgD* expression whereas its deletion in the STM4551 STM1987 double mutant did not have this effect (Fig. 5a) suggesting that STM3611 degrades the c-di-GMP synthesized by the GGDEF domain STM4551 and STM1987. The specificity of STM3611 towards STM1987 and STM4551 is consistent with the role of these proteins in motility regulation [40].

Deletion of the GGDEF-EAL protein STM1703 in the Δ4DGC mutant background invariably showed rdar morphotype formation and *csgD* expression as the STM1703 single mutant (Fig. 5b). Furthermore, combined deletion of STM1703 and the remaining diguanylate cyclases STM1283, STM2672, and AdrA did not diminish *csgD* expression below the level of the STM1703 mutant. This finding leads to the hypothesis that STM1703 degrades c-di-GMP produced from its own GGDEF domain, acting as a diguanylate cyclase locally while it might form a complex with a high affinity c-di-GMP receptor (Fig. 5b).

Deletion of the phosphodiesterase STM4264 in the Δ4DGC mutant enhanced rdar morphotype and CsgD expression to the level of the UMR1 wild type (Fig. 5c) suggesting an additional source of c-di-GMP to contribute to *csgD* expression. Stereomicroscopic visualization of the rdar morphotype of the STM4264 mutant with deletion of the four diguanylate cyclases (Δ4DGC) indicates a 3-D colony architecture different from UMR1 although CsgD expression is at almost similar levels indicating the distinct role of individual c-di-GMP turn over proteins in development of the rdar colony (Fig. 5d). Interestingly, 3-D architecture of *S. typhimurium* UMR1 and mutant strains is more pronounced on LB without salt agar plates as compared to LB without salt plates supplemented with Congo red and brilliant blue G (Fig. 5d, e), which indicates a negative effect of these dyes on rdar morphotype formation.

Deletion of STM1827 from single and double mutants of diguanylate cyclases did alter neither rdar morphotype nor *csgD* expression (Additional file 2: Figure S3) indicating that STM1827 contributes to degradation of the global c-di-GMP pool only to some extent.

### C-di-GMP signalling regulates *csgD* expression at multiple levels

We previously proposed transcriptional, posttranscriptional and posttranslational control of *csgD* expression by c-di-GMP [20]. Here, we investigated the target process of c-di-GMP mediated *csgD* expression under physiologically relevant changes in c-di-GMP levels in the Δ4DGC mutant as well as in STM4264 and STM1703 deletion mutants from transcriptional regulation to functionality of CsgD.

**Fig. 6:**
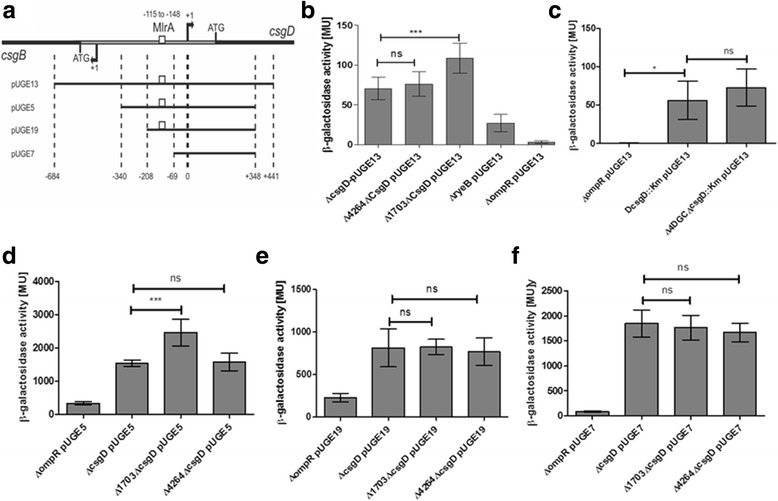
Effect of c-di-GMP on *csgD* transcription in the *S. typhimurium csgD* mutant background. **a** Schematic drawing of fusion constructs containing the *csgD* promoter region of different length. Transcriptional activity of the *csgD* promoter region was analyzed in pUGE13 [25, 36] (**b**) upon deletion of STM1703 and STM4264 and (**c**) upon deletion of four diguanylate cyclases (Δ4DGCs) compared to the respective Δ*csgD*::Km background The transcriptional activity was not affected in the Δ4DGC mutant whereas enhanced β-galactosidase activity was observed in *Δ1703* compared to Δ*csgD:101*. **d**-**f** Identification of the STM1703 regulatory region. As enhanced activity was observed only for pUGE5, but not for other fusions, STM1703 acts via the upstream region between nt −340 and −208. Controls were Δ*ryeB* with partial reduction of *csgD* transcription [28] and Δ*ompR* mutant as negative control. β-galactosidase measurements were done in duplicates using at least three technical replicates. Statistical significance is indicated by *P < 0.05 as compared to the respective *ΔcsgD* wild type control using unpaired *t*-test (Prism 5, GraphPad Software)

Using *csgD* promoter fusions to beta galactosidase that comprise the entire promoter region, UTR and part of the open reading frame from -684 to +441bp (Fig. 6a) indicated statistically significant enhancement of *csgD* promoter activity compared to the single *csgD* deletion background upon deletion of STM1703, whereas deletion of STM4264 tends to increase promoter activity (Fig. 6b). In contrast, the deletion of the 4 DGCs had no effect on *csgD* transcription (Fig. 6c). These results are consistent with previous reports of STM1703 to affect *csgD* transcription [20]. The c-di-GMP pool degraded by STM4264 and produced by 4 DGCs probably affects mainly posttranscriptional events beyond the fusion construct such as mRNA processing and stability.

To narrow down the c-di-GMP responsive regulatory region within the *csgD* upstream region in respect to STM1703, ß-galactosidase assays were performed with promoter fusions with subsequent extensions of the *csgD* promoter region (Fig. 6a) and [20, 23]. In summary, the results show that STM1703 acts on *csgD* expression in a distinct region, which could be traced between −340 and −208 upstream to the transcriptional start site of *csgD*.

**Fig. 7:**
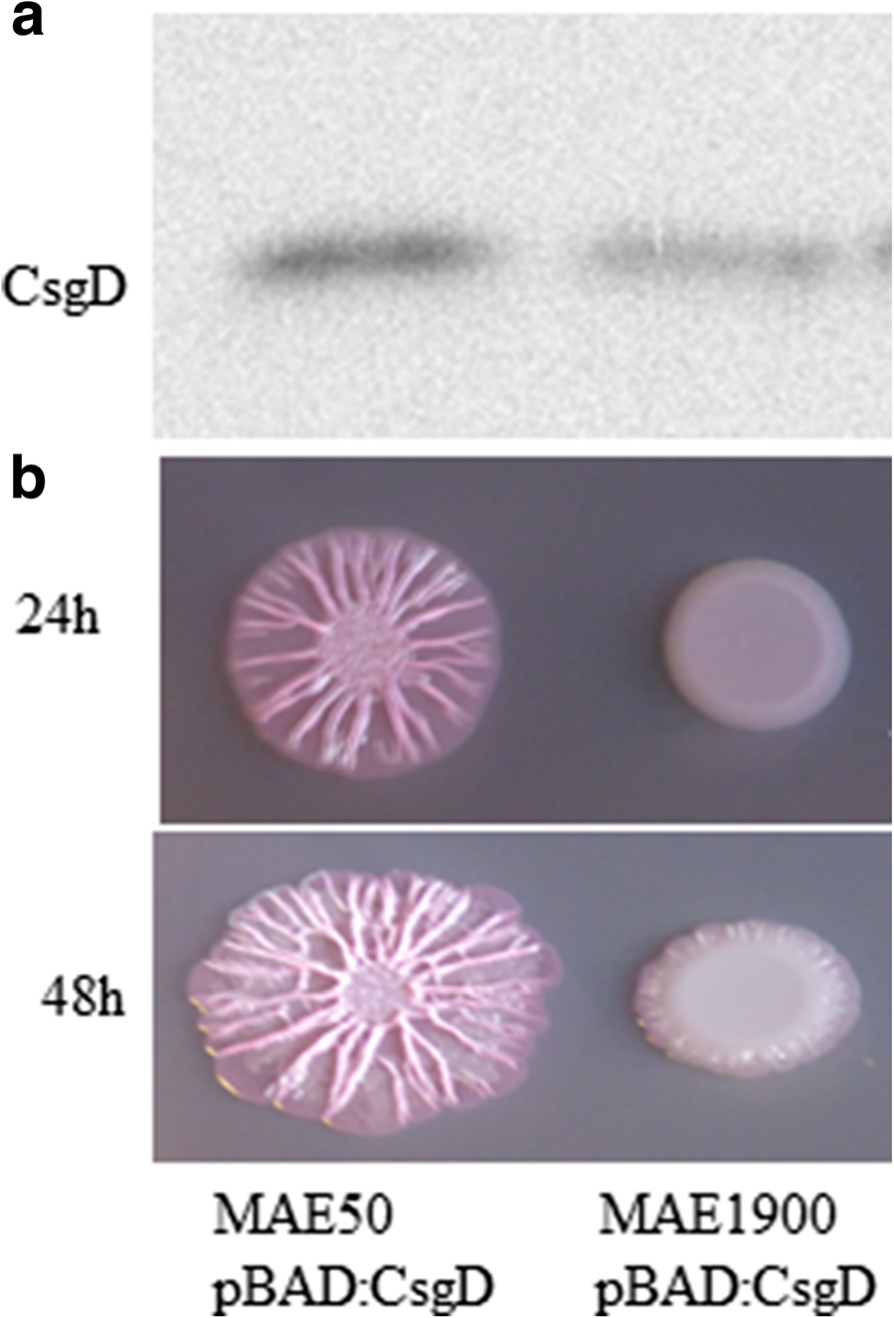
Overexpression of the PDE STM4264 from chromosome under *lacUV5* promoter suppresses CsgD production and rdar morphotype formation. CsgD expression (**a**) and rdar morphotype formation (**b**) upon overexpressing *csgD* from pBAD30 in *S. typhimurium* strain MAE1900 where STM4264 is expressed from the constitutive *lacUV5* promoter as compared to the Δ*csgD:101* deletion strain MAE50

**Fig. 8:**
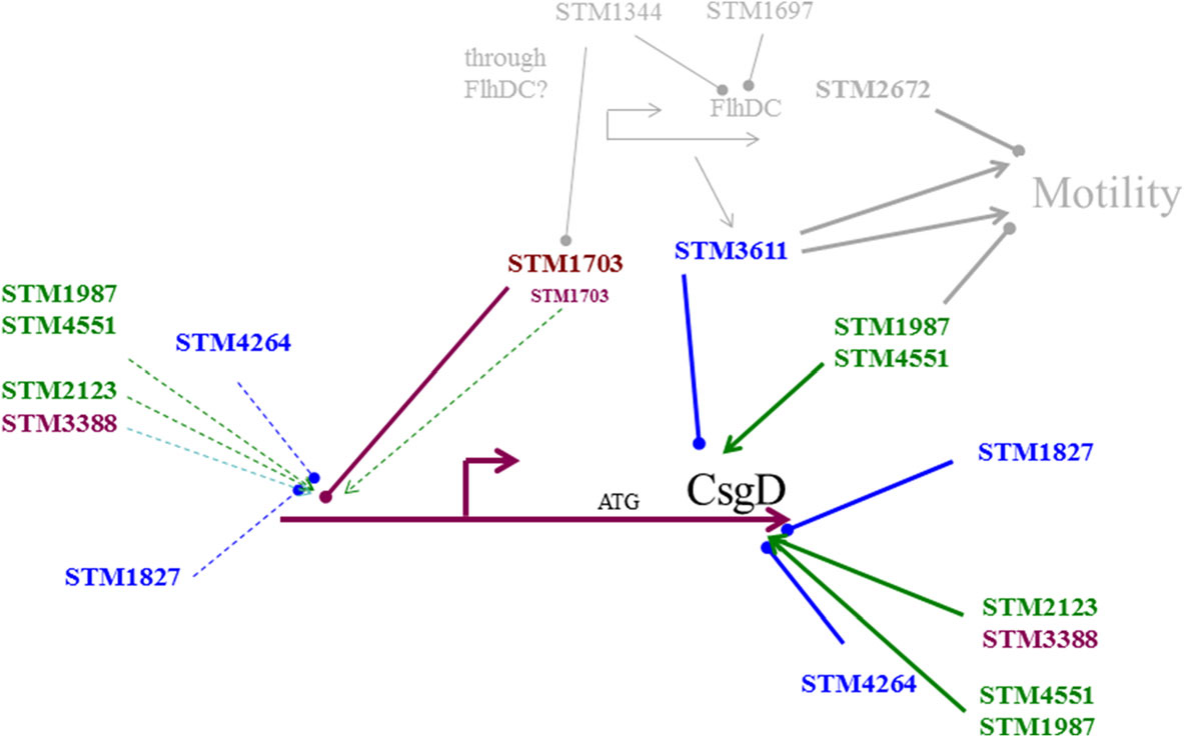
Schematic diagram showing the regulatory network of c-di-GMP signaling regulating *csgD* expression. At least three distinct groups of c-di-GMP turnover proteins regulate *csgD* expression. Regulation of *csgD* by STM1703 occurs on the transcriptional level, while the regulatory level by the other groups is unknown and drawn arbitrarily. The STM1987/STM4551/STM3611 group inversely regulates motility [40]. EAL-like proteins STM1344 and STM1697 affect the c-di-GMP signaling network through post-translational inhibition of FlhD_4_C_2_, the regulator of the flagellar cascade and regulation of STM1703 [41, 55]. Green represents a diguanylate cyclase, blue a phosphodiesterase and magenta represents a diguanylate cyclase/phosphodiesterase; light grey not directly investigated in this work

Investigating the expression of *csgD* from pBAD30 in different GGDEF/EAL mutant backgrounds did not reveal an effect of c-di-GMP (Additional file 2: Figure S5A). Assessment of CsgD functionality, assessed through its effect on *adrA* transcription in different mutant backgrounds did not show an effect of c-di-GMP (Additional file 2: Figure S5B). This finding indicates that the function of CsgD is not dependent on c-di-GMP levels. However, chromosomal over expression of STM4264 suppresses the expression of *csgD* (Fig. 7 and [31]). Cumulatively, these findings suggest that c-di-GMP enhances *csgD* expression by acting on multiple levels. Involvement of GGDEF/EAL domain proteins in regulation of *csgD* expression is summarized in Fig. 8.

## Discussion

Previously, we identified a role for GGDEF/EAL proteins in regulation of *csgD* expression and rdar morphotype formation in *S. typhimurium* [20]. In the present study, we showed that, in most instances, the catalytic activity of GGDEF/EAL domain proteins is required for the regulation of rdar morphotype and *csgD* expression. In addition, networks of corresponding c-di-GMP metabolizing proteins were identified.

GGDEF/EAL domain proteins can regulate biofilm formation through their enzymatic activities, but also, independently, through protein-protein interactions. For example, EAL domain proteins STM1697 and STM1344 are enzymatically inactive, but contribute to *csgD* and rdar morphotype expression by interacting with the FlhD_2_ subunit of FlhD_2_C_4_, the master regulator of flagella biogenesis in *S. typhimurium* [41, 42]. Similarly, GdpS, a GGDEF domain protein in *Staphylococcus aureus* and *Staphylococcus epidermidis*, does not exhibit diguanylate cyclase activity, but is nevertheless required for biofilm formation and expression of the extracellular matrix polysaccharide PAG (Poly N-acetyl glucosamine) [43, 44]. Moreover, the BLUF-EAL domain protein YcgF of *E. coli* does not exhibit phosphodiesterase activity, but contributes to expression of colonic acid and repression of curli fimbriae [45, 46]. Furthermore, GGDEF/EAL domain proteins regulate rdar formation and *csgD* expression through their enzymatic activities by c-di-GMP turnover with the exception of STM1703. This is in contrast to the previous preposition that *csgD* expression requires the GGDEF domain protein STM4551, but not its catalytic activity [39].

An additional role of the GGDEF proteins STM1987 and STM4551 in *csgD* expression has been identified, those proteins were previously known only to stimulate cellulose production [21].

BphG1, a GGDEF-EAL domain protein of *Rhodobacter sphaeroides,* ScrC of *Vibrio cholerae* and MSDGC1 of *Mycobacterium tuberculosis* are bi-functional GGDEF-EAL domain proteins [47, 48]. In this study, we confirm a bi-functional enzymatic activity of the GGDEF-EAL domain protein STM3388 *in vivo* through construction of mutant proteins.

Occurrence of multiple c-di-GMP metabolizing proteins in bacterial species raises the question of target specificity. Although eight of the GGDEF/EAL domain proteins are found to be involved in the regulation of *csgD* expression, indications for local regulation exists. Particularly, elevated level of *csgD* in the STM1703 mutant could not be restored upon the deletion of several diguanylate cyclases suggesting that the regulation of *csgD* by STM1703 can occur locally. This unconventional behavior of STM1703 led us to hypothesize that STM1703 forms a complex with a high affinity c-di-GMP receptor. Complex formation of GGDEF/EAL domain proteins with c-di-GMP receptors to regulate target processes locally occurs in *E. coli* where the c-di-GMP effector PNPase is physically associated with the diguanylate cyclase DosC and the phosphodiesterase DosP in the RNA degradosome [49]. Recently, the STM1703 homologue in *E. coli* has been proposed to function as a trigger protein, which senses and effectively degrades c-di-GMP produced upstream in the regulatory cascade thereby releasing inhibition of the diguanylate cyclase YdaM and the transcriptional regulator MlrA [50]. However, in *S. typhimurium*, a diguanylate cyclase corresponding to YdaM is not present. Also, the promoter upstream region that mediates STM1703 dependent *csgD* regulation from −208 to −340 does not correspond to the putative MlrA binding site, which we identified to be located at nts −115 to −148. Of note, we were unable to delete *mlrA* in *S. typhimurium* UMR1, but overexpression of MlrA showed the previously reported phenotype of CsgD upregulation [51].

On the other hand, the additive effect of the four diguanylate cyclases on down regulation of rdar morphotype and *csgD* expression in the deletion mutant of the phosphodiesterase STM4264 suggests a global impact of c-di-GMP on *csgD* expression regulated by this phosphodiesterase. This is consistent with c-di-GMP levels to be substantially elevated upon deletion of STM4264, whereas deletion of STM1703 leads only to a marginal increase of c-di-GMP, despite higher *csgD* expression in the STM1703 mutant [49]. Similarly, VpsT, a member of the LuxR-CsgD family in *V. cholerae*, is regulated by a global pool of c-di-GMP assembled by at least five diguanylate cyclases [52].

VpsT is not only regulated by c-di-GMP at multiple levels, but is also able to bind c-di-GMP to efficiently regulate transcription of target genes [53, 54]. In contrast, CsgD from Enterobacteriaceae lacks the c-di-GMP binding motif [29]. The complex regulation of *csgD* expression by c-di-GMP signalling suggests involvement of more than one c-di-GMP effector in modulation of *csgD* expression. Identification of the c-di-GMP receptors and elucidation of molecular mechanisms leading to c-di-GMP mediated regulation of *csgD* expression is an interesting subject for follow-up studies.

## Conclusions

Several GGEDEF/EAL proteins have recently been shown to regulate target processes through protein-protein interactions. Our findings propose that regulation of *csgD* expression and rdar biofilm development by GGDEF/EAL domain proteins is mainly, but not exclusively, mediated through the enzymatic activities of the proteins. The diguanylate cyclase activity of GGDEF domain proteins contributes to promote *csgD* expression. Thereby, the GGDEF proteins STM4551, STM1987, STM3388 and STM2123 have an additive effect on the promotion of *csgD* expression. Moreover, *csgD* is regulated by c-di-GMP signalling at multiple levels. The GGDEF/EAL protein STM 1703 suppresses the transcription of *csgD*, whereas EAL domain STM 4264 suppresses *csgD* by acting on post-transcriptional events.

C-di-GMP signalling has recently emerged as an important intracellular tool to promote biofilm formation in a concerted action in many pathogenic and environmental bacterial species. Our findings extend the understanding of the mechanisms of the regulation of target processes by c-di-GMP signalling in *S. typhimurium*.

## Additional files

Additional file 1: Table S1. Strains and plasmids, **Table S2.** Primers and references to supplementary material. (DOCX 40 kb)

Additional file 2: Figure S1. Complementation of rdar morphotype and *csgD* expression by cyclic di-GMP turnover proteins. **Figure S2.** CsgD levels and rdar morphotype formation of *S. typhimurium* UMR1 upon expression of the GGDEF-EAL protein STM1703 and its catalytic mutants. **Figure S3.** STM1827 regulates rdar morphotype and *csgD* expression by degrading the global pools of c-di-GMP. **Figure S4.** Enhanced rdar morphotype in STM4264 and STM1703 mutants is dependent on the transcriptional regulators RpoS and OmpR. **Figure S5.** Effect of c-di-GMP signalling on translation and functionality of CsgD. **Figure S6.** Schematic representation of GGDEF/EAL proteins and mutants used in the study. (DOCX 1739 kb)
