# Supplementary material for: Detailed analysis of c-di-GMP mediated regulation of csgD expression in Salmonella typhimurium
Source: BMC Microbiol. 2017 Feb 2;17:27. doi: 10.1186/s12866-017-0934-5 (PMC5289004; doi:10.1186/s12866-017-0934-5)
Supplement: Supplementary file 1 — Strains and plasmids, Table S2. Primers and references to supplementary material. (DOCX 40 kb) [file 12866_2017_934_MOESM1_ESM.docx]

# Detailed analysis of c-di-GMP mediated regulation of *csgD* expression in *Salmonella typhimurium*

# Irfan Ahmad^1^, Annika Cimdins^1^, Timo Beske^1^, and Ute Römling^1^*

^1^Department of Microbiology, Tumor and Cell Biology, Karolinska Institutet, Stockholm, Sweden

* For correspondence [ute.romling@ki.se](mailto:ute.romling@ki.se)

**Table S1 Strains and plasmids used in this study**

| Strain and Plasmid | Genotype | Source |
| --- | --- | --- |
| **Strains** | | |
| UMR1 | ATCC14028-1s Nal^r^ | [[1](#_ENREF_1)] |
| MAE263 | UMR1 STM4551::Cm^r^ | [[2](#_ENREF_2)] |
| MAE259 | UMR1 STM1987::Cm^r^ | [[2](#_ENREF_2)] |
| MAE121 | UMR1 STM3388::Km^r^ | [[2](#_ENREF_2)] |
| MAE272 | UMR1 STM2123::Cm^r^ | [[2](#_ENREF_2)] |
| MAE420 | UMR1 STM3611::Cm^r^ | [[3](#_ENREF_3)] |
| MAE425 | UMR1 STM4264::Cm^r^ | [[3](#_ENREF_3)] |
| MAE282 | UMR1 STM1703::Cm^r^ | [[3](#_ENREF_3)] |
| MAE422 | UMR1 STM1827::Cm^r^ | [[3](#_ENREF_3)] |
| MAE1543 | UMR1 STM4264_E313A_ | This study |
| MAE1489 | UMR1 STM1987::Cm^r^ STM4551::101 | [[4](#_ENREF_4)] |
| MAE275 | UMR1 STM3388::Km^r^ STM2123::cm^r^ | [[2](#_ENREF_2)] |
| MAE1547 | UMR1 STM2123::Cm^r^ STM1987::101 STM4551::101 | This study |
| MAE1549 | UMR1 STM2123::101 STM1987::101 STM4551::101 | This study |
| MAE1881 | UMR1 STM1827::101 STM2123::Cm^r^ | This study |
| MAE1880 | UMR1 STM1827::101 STM3388::Km^r^ |  |
| MAE1879 | UMR1 STM1827::Cm^r^ STM4551::101 STM1987::101 | This study |
|  | UMR1 STM3611::101 STM3388::Km^r^ | This study |
| MAE1521 | UMR1 STM3611::Cm^r^ STM4551::101 STM1987::101 | [[4](#_ENREF_4)] |
| MAE1821 | UMR1 STM4264::101 STM2123::Cm^r^ STM3388::Cm^r^ | This study |
| MAE1507 | UMR1 STM4264::Cm^r^ STM4551::101 STM1987::101 | [[4](#_ENREF_4)] |
| MAE1852 | UMR1 STM4264::Cm^r^ STM4551::101 STM1987::101 STM2123::101 STM 3388::101 | This study |
| MAE1853 | UMR1 STM 1703::101 STM 4551::101 STM1987::101 STM2123::101 STM3388::101 | This study |
| MAE448 | UMR1 STM1703::101 *adrA*:103 | [[3](#_ENREF_3)] |
| MAE431 | UMR1 STM2672::Cm^r^ STM1703::101 | [[3](#_ENREF_3)] |
| MAE1876 | UMR1 STM2672::101 STM 1703::101 | This study |
| MAE1877 | UMR1 STM1283::Cm^r^ STM2672::101 STM1703::101 | This study |
| MAE1878 | UMR1 STM1283::Cm^r^ STM2672::101 STM1703::101 *adrA*::101 | This study |
| MAE50 | UMR1 Δ*csgD*::101 | [[5](#_ENREF_5)] |
| MAE52 | UMR1 *pcsgD1* | [[6](#_ENREF_6)] |
| MAE437 | UMR1 STM 4264::Cm Δ*csgD*::101 | [[4](#_ENREF_4)] |
| MAE432 | UMR1 STM 1703::Cm^r^ Δc*sgD*::101 | [[3](#_ENREF_3)] |
| MAE1854 | UMR1 *ΔcsgD*::Km^r^ STM2123::101 STM3388::101 STM4551::101 STM 1987::101 | This study |
| MAE28 | UMR1 Δ*csgD*::Km^r^ | [[3](#_ENREF_3)] |
| MAE1861 | UMR1 Δ*csgD*::101 *adrA*::MudJ | This study |
| MAE1864 | UMR1 STM4264::Cm^r^ Δ*csgD*::101 *adrA*::Mudj | This study |
| MAE1862 | UMR1 STM1703::Cm^r^ Δ*csgD*::101 *adrA*::Mudj | This study |
| MAE1866 | UMR1 Δ*ryeB*::Km^r^ Δ*csgD*::101 | This study |
| MAE1570 | UMR1 ΔryeB::Km^r^ | [[7](#_ENREF_7)] |
| MAE1871 | UMR1 Δ*ompR*::Cm^r^ | This study |
| MAE1867 | UMR1 Δ*ompR*::Cm^r^ STM4264::101 | This study |
| MAE1868 | UMR1 Δ*ompR*::Cm^r^ STM1703::101 | This study |
| MAE1573 | UMR1 Δ*rpoS*::Cm^r^ | [[7](#_ENREF_7)] |
| MAE1869 | UMR1 Δ*rpoS*::Cm^r^ STM4264::101 | This study |
| MAE1870 | UMR1 ΔrpoS::Cm^r^ STM1703::101 | This study |
| MAE1490 | UMR1 Δ4551::101 STM1987::101 | [[4](#_ENREF_4)] |
| MAE462 | UMR 1 STM1827::101 | This study |
| Adra1f | adrA101::MudJ | [[2](#_ENREF_2)] |
| ME490 | UMR1 STM1703 npt lacI PlacUV5 STM4264 | [[3](#_ENREF_3)] |
| MAE1900 | MAE50 lacI PlacUV5 STM4264:Km | This study |
| **Plasmids** |  |  |
| pBAD30 | Arabinose regulated expression vector, Amp^r^ | [[8](#_ENREF_8)] |
| pBAD30::4551 | pBAD30 with *SacI/HindIII*-ligated STM4551-6XHis fragment | [[4](#_ENREF_4)] |
| pBAD30::4551_E267A_ | pBAD30::4551 With STM4551_E267A_ (GGE_267_EF) motif changed to GGAEF | [[4](#_ENREF_4)] |
| pRGS1 | pBAD30 with *XbaI/SphI*-ligated STM3611-6XHis fragment | [[9](#_ENREF_9)] |
| pBAD30::3611_E136A_ | pRGS1 with STM3611_E136A_ | [[9](#_ENREF_9)] |
| pBAD30::3611_K179A_ | pRGS1 with STM3611_K79A_ | [[4](#_ENREF_4)] |
| pRGS2 | pBAD30 with *XbaI/HindIII*-ligated STM1827-6XHis fragment | [[9](#_ENREF_9)] |
| pBAD30::1827_E302A_ | pRGS2 with STM1827_E302A_ | This study |
| pBAD30::1827_K339A_ | pRGS2 with STM1827_K339A_ | This study |
| pRGS19 | pBAD30 with *XbaI/HindIII*-ligated STM3388-6XHis fragment | [[2](#_ENREF_2)] |
| pBAD30::3388_D342A_ | pRGS19 with STM3388_D342_ (GGD_342_EF) motif changed to GGAEF | This study |
| pBAD30::3388_E467A_ | pRGS19 with STM3388_E467A_ (E_467_AL) motif changed to AAL | This study |
| pBAD30::3388_E644A_ | pRGS19 with STM3388_E644A_ | This study |
| pBAD30::2123 | pBAD30 with *XbaI/HindIII*-ligated STM2123-6XHis fragment | This study |
| pBAD30::2123_D651A_ | pBAD30::2123 with STM2123_D651A_ (GGD_651_EF) motif changed to GGAEF | This study |
| pRGS25 | pBAD30 with *XbaI/HindIII*-ligated STM1703-6XHis fragment | [[9](#_ENREF_9)] |
| pBAD30::1703_D313A_ | pRGS25 with STM1703_D313A_ (GGD_313_EF) motif changed to GGAEF | This study |
| pBAD30::1703_E437A_ | pRGS25 with STM1703_E437A_ (EAL) motif changed to AAL | This study |
| pBAD30::1703_E527A_ | pRGS25 with STM1703_E527_  changed to STM1703_A527_ | This study |
| pBAD30::1703_K578A_ | pRGS25 with STM1703_K578_  changed to STM1703_A578_ | This study |
| pBAD30::1703_E613A_ | pRGS25 with STM1703_E613_  changed to STM1703_A613_ | This study |
| pBAD30::2123_R640A_ | pBAD30::2123 with STM2123_R640_ (R XXD) motif changed to STM2123_A640_ |  |
| pUGE5 | pQF50 containing fragment +348/-340 of *PcsgD* | [[10](#_ENREF_10)] |
| pUGE7 | pQF50 containing fragment +348/-69 of *PcsgD* | [[10](#_ENREF_10)] |
| pUGE13 | pQF50 containing fragment +441/-684 of *PcsgD* | [[11](#_ENREF_11)] |
| pUGE19 | pQF50 containing fragment +348/-208 of *PcsgD* | [[11](#_ENREF_11)] |
| pUMR15 | pBAD30 containing CsgD | [[5](#_ENREF_5)] |
| pQF50 | promoter less *lacZ* gene, RO1600/MB1, Amp^r^ | [[12](#_ENREF_12)] |

**Table S2 Primers used in study**

| **Primer** | **Sequence** |
| --- | --- |
| STM1703_D313A_ F | CTGGGGGGAGCTGAATTCATTG |
| STM1703_D313A_ R | CAATGAATTCAGCTCCCCCCAG |
| STM1703_K578A_ F | ATGCCGTTGCACTGGATCAG |
| STM1703_K578A_ R | CTGATCCAGTGCAACGGCAT |
| STM703E_527A_ F | GCCCGATCGATGTCGCGCTAACGGAAAGCTGCC |
| STM1703_E527A_ R | GGCAGCTTTCCGTTAGCGCGACATCGATCGGGC |
| STM1703_E613A_ F | CGCTAAATTTACAAGTGATTGCCGCAGGTGTAGAAAATGC |
| STM1703_E613A_ R | GCATTTTCTACACCTGCGGCAATCACTTGTAAATTTAGCG |
| STM3388 _E467A_ F | CTATTGTAGGATTTGCTGCCCTGTTGCGCT |
| STM3388 _E467A_ R | AGCGCAACAGGGCAGCAAATCCTACAATAG |
| STM3388 _E644A_ R | CATTCTCTACCCCAGCTGCCACAATTTGCAG |
| STM3388 _E644A_ F | CTGCAA CTGCAAATTGTGGCAGCTGGGGTAGAGAATG |
| STM3388 _D342A_ F | GCGCGGATTGGCGGCGCTGAATTTGTGCTGCTTG |
| STM3388 _D342A_ R | CAAGCAGCACAAATTCAGCGCCGCCAATCCGCGC |
| STM2123 _D651A_ F | CCGTCTGGGTGGCGCTGAGTTTGGCCTGTT |
| STM2123 _D651A_ R | AACAGGCCAAACTCAGCGCCACCCAGACGG |
| STM1827 _E302A_ F | CAGTGTACCGGCGTTGCCATCCTGTTACGCTGG |
| STM1827 _E302A_ R | CCAGCGTAACAGGATGGCAACGCCGGTACACTG |
| 4264-insert-cm- forw | CAGCCGATCATTGATATCCGAAACGGGACATGTGTAGGTGCGCCTTAC GCCCCGCGCTGC |
| 4264-insert-cm-rev | CTCATCACCGGACCGTGATAGCCCGGCCAACGCAATAGCGCTAGACTAT ATTACCCTGTT |
| 4264-303A-mut-scarless forw | CAGCCGATCATTGATATCCGAAACGGGACATGTGTAGGTGCCGCGGCC TATTGCGTTGGCCGGGCTATCACGGTCCGGTGATGAG |
| 4264-E303A-mut-scarless Rev | CTCATCACCGGACCGTGATAGCCCGGCCAACGCAATAGCGCCGCGGC ACCTACACATGTCCCGTTTCGGATATCAATGATCGGCTG |
| 0mpR ko forw | ATGCAAGAGAATTATAAGATTCTGGTGGTTGATGACGATAGTGTAGG CTGGAGCTGCTTC |
| ompR ko rev | TCATGCTTTAGAACCGTCCGGTACAAAGACGTAGCCCAGGCATATGT ATCCTCCTTAGT |
| ompR control for | GTTTGAGTGTTTCGTACCC |
| ompR control rev | TGACGATGAGCAACAGCGT |

REFERENCES

1. Römling U, Bian Z, Hammar M, Sierralta WD, Normark S: **Curli fibers are highly conserved between Salmonella typhimurium and Escherichia coli with respect to operon structure and regulation**. *J Bacteriol* 1998, **180**:722-731.

2. Kader A, Simm R, Gerstel U, Morr M, Römling U: **Hierarchical involvement of various GGDEF domain proteins in rdar morphotype development of *Salmonella enterica* serovar Typhimurium**. *Mol Microbiol* 2006, **60**:602-616.

3. Simm R, Lusch A, Kader A, Andersson M, Römling U: **Role of EAL-containing proteins in multicellular behavior of *Salmonella enterica* serovar Typhimurium**. *J Bacteriol* 2007, **189**:3613-3623.

4. Ahmad I, Lamprokostopoulou A, Le Guyon S, Streck E, Peters V, Barthel M, Hardt W-D, Römling U: **Complex c-di-GMP signaling networks mediate the transition between virulence properties and biofilm formation in *Salmonella enterica* serovar Typhimurium**. *PloS One* 2011, **6**:e28351.

5. Römling U, Rohde M, Olsen A, Normark S, Reinköster J: **AgfD, the checkpoint of multicellular and aggregative behaviour in *Salmonella typhimurium* regulates at least two independent pathways**. *Mol Microbiol* 2000, **36**:10-23.

6. Römling U, Sierralta WD, Eriksson K, Normark S: **Multicellular and aggregative behaviour of *Salmonella typhimurium* strains is controlled by mutations in the *agfD* promoter**. *Mol Microbiol* 1998, **28**:249-264.

7. Monteiro C, Papenfort K, Hentrich K, Ahmad I, Le Guyon S, Reimann R, Grantcharova N, Romling U: **Hfq and Hfq-dependent small RNAs are major contributors to multicellular development in Salmonella enterica serovar Typhimurium**. *RNA biology* 2012, **9,**.

8. Guzman LM, Belin D, Carson MJ, Beckwith J: **Tight regulation, modulation, and high-level expression by vectors containing the arabinose PBAD promoter**. *J Bacteriol* 1995, **177**:4121-4130.

9. Simm R, Morr M, Kader A, Nimtz M, Römling U: **GGDEF and EAL domains inversely regulate cyclic di-GMP levels and transition from sessility to motility**. *Mol Microbiol* 2004, **53**:1123-1134.

10. Gerstel U, Park C, Römling U: **Complex regulation of csgD promoter activity by global regulatory proteins**. *Mol Microbiol* 2003, **49**:639-654.

11. Gerstel U, Kolb A, Römling U: **Regulatory components at the csgD promoter – additional roles for OmpR and integration host factor and role of the 5′ untranslated region**. *FEMS Microbiology Letters* 2006, **261**:109-117.

12. Farinha MA, Kropinski AM: **Construction of broad-host-range plasmid vectors for easy visible selection and analysis of promoters**. *J Bacteriol* 1990, **172**:3496-3499.
