# Supplementary material for: Detailed analysis of c-di-GMP mediated regulation of csgD expression in Salmonella typhimurium
Source: BMC Microbiol. 2017 Feb 2;17:27. doi: 10.1186/s12866-017-0934-5 (PMC5289004; doi:10.1186/s12866-017-0934-5)
Supplement: Supplementary file 2 — Complementation of rdar morphotype and csgD expression by cyclic di-GMP turnover proteins. Figure S2. CsgD levels and rdar morphotype formation of S. typhimurium UMR1 upon expression of the GGDEF-EAL protein STM1703 and its catalytic mutants. Figure S3. STM1827 regulates rdar morphotype and csgD expression by degrading the global pools of c-di-GMP. Figure S4. Enhanced rdar morphotype in STM4264 and STM1703 mutants is dependent on the transcriptional regulators RpoS and OmpR. Figure S5. Effect of c-di-GMP signalling on translation and functionality of CsgD. Figure S6. Schematic representation of GGDEF/EAL proteins and mutants used in the study. (DOCX 1739 kb) [file 12866_2017_934_MOESM2_ESM.docx]

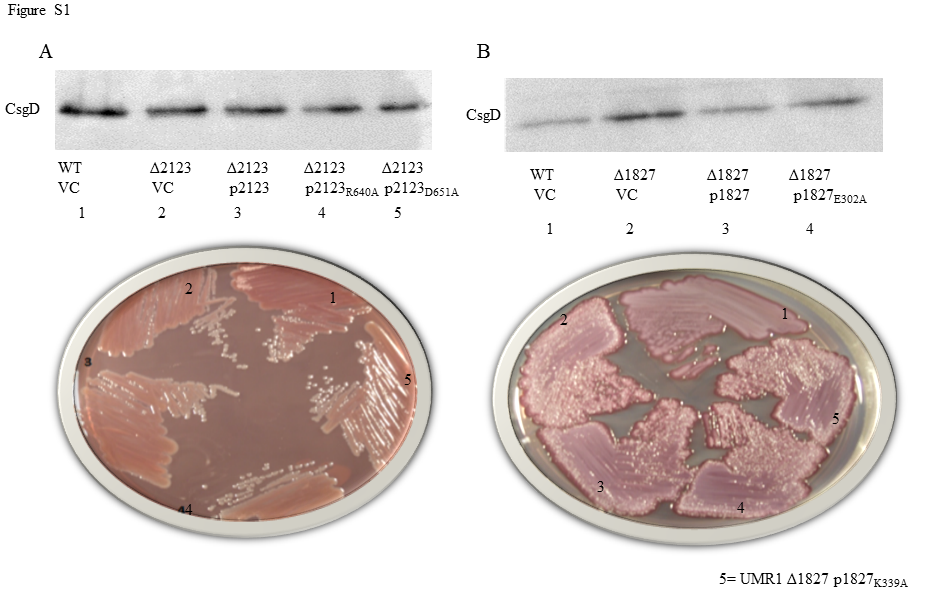


**Figure S1 Complementation of rdar morphotype and *csgD* expression by cyclic di-GMP turnover proteins.** GGDEF domain protein STM2123 (A) and EAL domain protein STM1827 expressing wild type and catalytic mutant proteins in the respective *S. typhimurium* deletion mutant strains. Cells were grown on LB without salt agar plates supplemented with Ampicillin (100 μg ml^−1^) and 0.1% L-Arabinose for 24 h at 28°C to investigate rdar morphotype and CsgD levels. Captions VC= Vector control pBAD30, WT= Wild Type (UMR1.


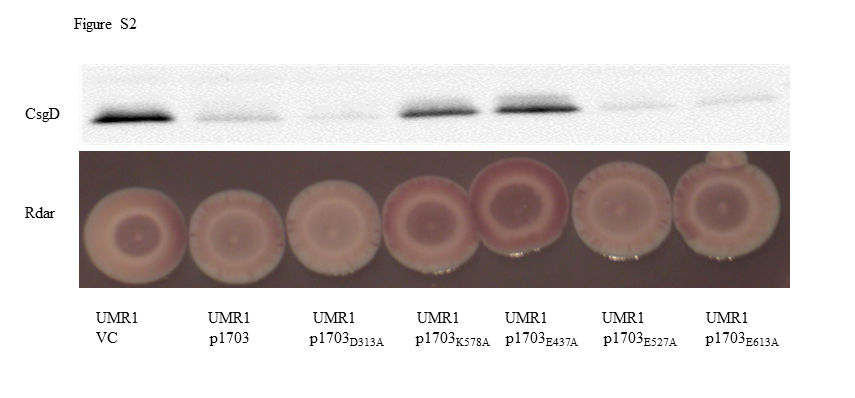


**Figure S2 CsgD levels and rdar morphotype formation of *S. typhimurium* UMR1 upon expression of the GGDEF-EAL protein STM1703 and its catalytic mutants**. The STM1703_D313A_ variant suppresses *csgD* expression more efficiently than the wild type protein suggesting diguanylate cyclase activity of the protein, whereas the catalytic EAL mutant STM1703_E437A_ promote biofilm formation in UMR1. Captions 1= UMR1 VC, 2= UMR1 p1703, 3= UMR1 p1703_D313A_, 4= UMR1 p1703_K578A_, 5= UMR1 p1703_E437A_, 6= UMR1 p1703_E527A_, 7= UMR1 p1703_E613A._


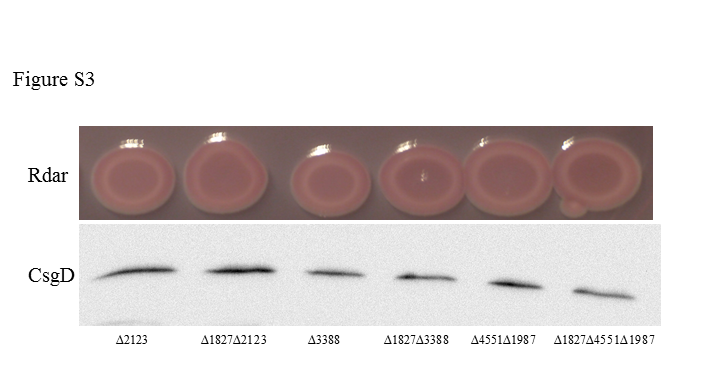


**Figure S3 STM1827 regulates rdar morphotype and *csgD* expression by degrading the global pools of c-di-GMP.** Rdar morphotype formation and CsgD levels upon deletion of the EAL domain protein STM1827 in the background of different GGDEF protein deletion strains of *S. typhimurium* UMR1


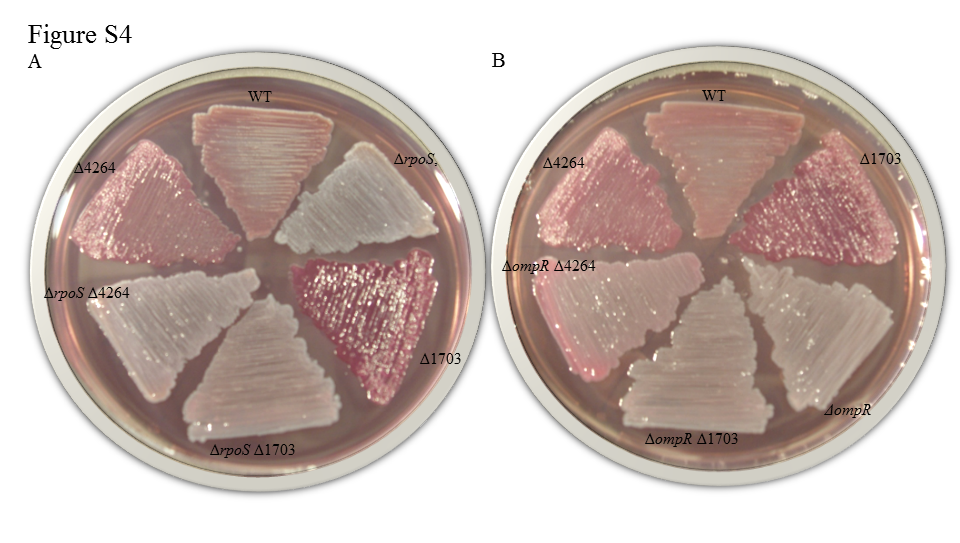


**Figure S4 Enhanced rdar morphotype in STM4264 and STM1703 mutants is dependent on the transcriptional regulators RpoS and OmpR.** Rdar morphotype formation upon deletion of the transcriptional regulator RpoS (A) and the transcriptional regulator OmpR (B) from the STM4264 and STM1703 deletion mutants of *S. typhimurium* UMR1.


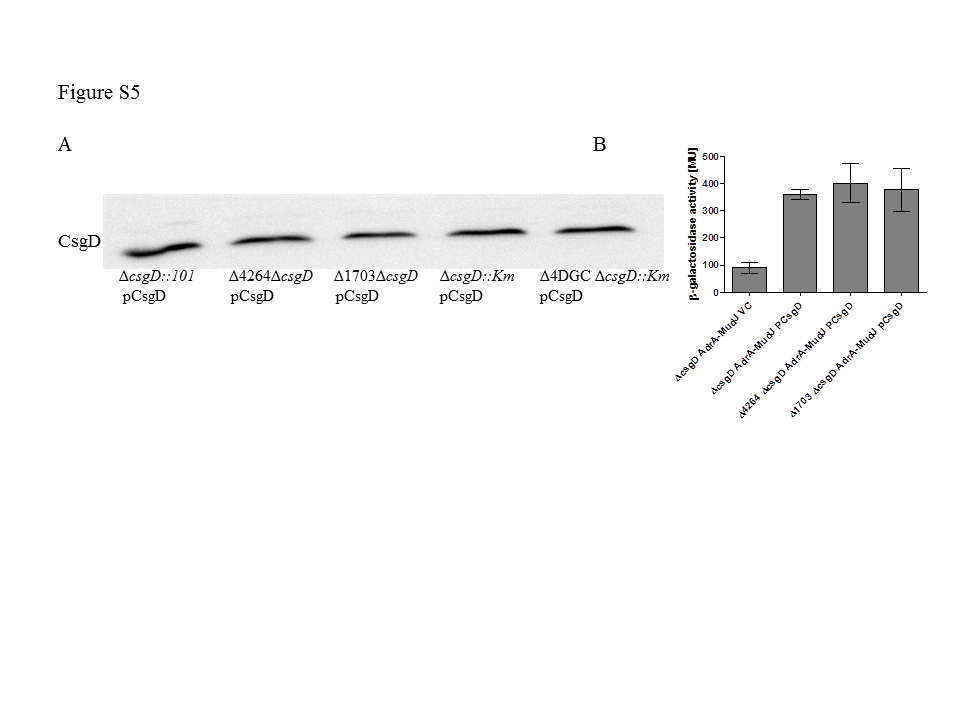


**Figure S5 Effect of c-di-GMP signalling on translation and functionality of CsgD.** (A) Effect of c-di-GMP levels on CsgD expression from an arabinose inducible promoter in *S. typhimurium* UMR1 compared to its STM4264, STM1703 and Δ4DGC deletion mutants. CsgD expression from pBAD30 was estimated by western blot analysis. CsgD levels were not altered in STM4264 and STM1703 mutants. The respective *csgD* deletion background was either MAE50 (Δ*csgD)* or MAE28 (*ΔcsgD::Km*). Captions pCsgD = CsgD cloned in the vector pBAD30.

(B) Effect of CsgD on transcription of *adrA* under high c-di-GMP levels. Transcriptional activity of *adrA* (measured as β-galactosidase activity) from transcriptional fusion of *adrA* with *MudJ* in the strain AdrA1f (AdrA:103:MudJ). Deletion of STM4264 and STM1703 did not alter *adrA* transcriptional activity upon concurrent *csgD* expression from plasmid in *S. typhimurium* UMR1. Cells were grown for 24 hours at 28°C on LB without salt plates.


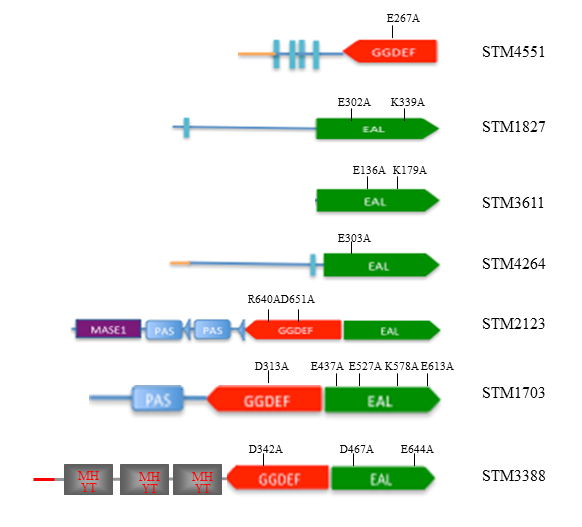


**Figure S6 Schematic representation of GGDEF/EAL proteins and mutants used in the study.**
